# Long-term neurodevelopment outcomes of hand, foot and mouth disease inpatients infected with EV-A71 or CV-A16, a retrospective cohort study

**DOI:** 10.1080/22221751.2021.1901612

**Published:** 2021-03-29

**Authors:** Lu Liang, Yibing Cheng, Yu Li, Qing Shang, Jiao Huang, Caiyun Ma, Shuanfeng Fang, Lu Long, Chongchen Zhou, Zhiping Chen, Peng Cui, Nan Lv, Pu Lou, Yajie Cui, Saraswathy Sabanathan, H. Rogier van Doorn, Rongsheng Luan, Lance Turtle, Hongjie Yu

**Affiliations:** a West China School of Public Health and West China Fourth Hospital, Sichuan University, Chengdu, People’s Republic of China; b Children’s Hospital Affiliated to Zhengzhou University, Henan Children’s Hospital, Zhengzhou, People’s Republic of China; c Division of Infectious Disease, Key Laboratory of Surveillance and Early-warning on Infectious Disease, Chinese Center for Disease Control and Prevention, Beijing, People’s Republic of China; d Department of Epidemiology and Biostatistics, State Key Laboratory of Environmental Health (Incubation), School of Public Health, Tongji Medical College, Huazhong University of Science and Technology, Wuhan, People’s Republic of China; e School of Public Health, Fudan University, Key Laboratory of Public Health Safety, Ministry of Education, Shanghai, People’s Republic of China; f Oxford University Clinical Research Unit, Ha Noi, Viet Nam; g Nuffield Department of Medicine, Centre for Tropical Medicine and Global Health, Oxford University, Oxford, UK; h NIHR Health Protection Research Unit for Emerging and Zoonotic Infections, Institute of Infection, Veterinary and Ecological Sciences University of Liverpool, Liverpool, UK; i Tropical & Infectious Disease Unit, Royal Liverpool University Hospital (member of Liverpool Health Partners), Liverpool, UK

**Keywords:** HFMD, EV-A71, CV-A16, outcomes, respiratory function

## Abstract

Hand, foot and mouth disease (HFMD) is a common infectious disease in western Asia area and the full range of the long-term sequelae of HFMD remains poorly described. We conducted a retrospective hospital-based cohort study of HFMD patients with central nervous system (CNS) complications caused by EV-A71 or CV-A16 between 2010 and 2016. Patients were classified into three groups, including CNS only, autonomic nervous system (ANS) dysregulation, and cardiorespiratory failure. Neurologic examination, neurodevelopmental assessments, Magnetic Resonance Imaging (MRI) and lung function, were performed at follow up. Of the 176 patients followed up, 24 suffered CNS only, 133 ANS dysregulation, and 19 cardiorespiratory failure. Median follow-up period was 4.3 years (range [1.4–8.3]). The rate of neurological abnormalities was 25% (43 of 171) at discharge and 10% (17 of 171) at follow-up. The rates of poor outcome were significantly different between the three groups of complications in motor (28%, 38%, 71%) domain (p=0.020), but not for cognitive (20%, 24%, 35%), language (25%, 36%, 41%) and adaptive (24%, 16%, 26%) domains (*p* = 0.537, *p* = 0.551, *p* = 0.403). For children with ventilated during hospitalization, 41% patients (14 of 34) had an obstructive ventilatory defect, and one patient with scoliosis had mixed ventilatory dysfunction. Persistent abnormalities on brain MRI were 0% (0 of 7), 9% (2 of 23) and 57% (4 of 7) in CNS, ANS and cardiorespiratory failure group separately. Patients with HFMD may have abnormalities in neurological, motor, language, cognition, adaptive behaviour and respiratory function. Long-term follow-up programmes for children’s neurodevelopmental and respiratory function may be warranted.

## Introduction

Hand, foot and mouth disease (HFMD) is a common infectious disease mainly affecting children under 5 years [1,2]. From 2008 to 2017 in mainland China, enterovirus A71 (EV-A71) and coxsackievirus (CV) A16 were the main pathogens causing HFMD, comprising 43.3% and 23.1% of laboratory-confirmed cases respectively [3]. Most HFMD cases are mild, but some rapidly develop severe neurologic and cardiopulmonary complications which can be fatal [4,5]. Several small size studies from mainland China, Taiwan China and Australia identified that children infected with EV-A71 can suffer from long-term sequelae, for example, long-term cognitive and motor dysfunction [6–9]. A large population-based study also showed that enterovirus infection is associated with speech and language impairment in children [10]. However, the full range of the long-term sequelae caused by EV-A71 or CV-A16 remains poorly described.

Adaptive behaviour is the age-appropriate ability of independent living and functioning well in daily life across conceptual, social, and practical domains and is as important as intellectual functioning in assessing intellectual disability [11], but formal adaptive behaviour assessments have been lacking in previous HFMD studies. Small follow-up study reported that patients with pulmonary edema had severe respiratory failure [7], but lacked details about impaired respiratory function after mechanical ventilation. Respiratory function in childhood is an important indicator of personal health and types and degrees of impaired respiratory function have different impacts on adult respiratory function [12]. Although the studies reported that lesions in the brainstem or spinal cord may be useful for predicting prognosis by Magnetic resonance imaging (MRI) [9,13], whether such MRI changes resolve or persist over time, and their relationship with long-term outcome, remain unclear.

Because of the plasticity of brain development in early childhood, identifying outcomes offers an opportunity for intervention [14,15]. As more patients survive HFMD, understanding the long-term outcomes is important for case management in the acute illness, follow-up programmes after the acute illness and evaluation of disease burden. This is one of the few up-to-date studies of long-term outcome following HFMD in mainland China, an area of high HFMD prevalence. We aimed to comprehensively evaluate long-term neurological and neurodevelopmental outcomes, along with MRI changes over time and lung function after mechanical ventilation according to clinical complications during acute HFMD. In addition, we explored the risk factors for poor outcomes in children with EV-A71 or CV-A16 infection.

## Methods

### Study participants

In this retrospective cohort study, HFMD patients who were treated in the Henan Children’s Hospital between January 2010 and December 2016 were identified. The Henan Children’s Hospital is the largest pediatric hospital in Henan province with 2,200 beds and is the designated referral hospital for severe HFMD.

HFMD patients who tested positive for either EV-A71 or CV-A16 virus by real-time RT–PCR, and who were recorded as having central nervous system (CNS) complications during hospitalization by reviewing the medical charts, and who survived the hospitalization, were included. CNS complications include meningitis, encephalitis, brainstem encephalitis, acute flaccid paralysis and encephalomyelitis, as defined by World Health Organization (WHO) guideline [16]. Patients with either preterm birth (defined as delivered before 37 weeks of gestation), or any prior chronic respiratory, cardiac illness, or previous pediatric intensive care unit (ICU) admission/ventilation, or prior learning disability, neurological regression or prior delayed development before the onset of HFMD – were excluded. Patients who could subsequently not be contacted were also excluded.

Among the enrolled subjects, we retrospectively collected clinical data during the acute illness from clinical records, including demographics, clinical course, treatments, complications, neurological examination at discharge and MRI findings in radiology reports using a standardized form. We divided the patients into three groups according to their complications during the acute disease: those with CNS complications only, or those with autonomic nervous system (ANS) dysregulation following CNS involvement, or those with cardiorespiratory failure (including pulmonary edema, pulmonary hemorrhage) following CNS involvement [16]. Patients were categorized as ANS dysfunction group when the patients exhibited any of the following features: presence of cold sweating, mottled skin, respiratory abnormalities (tachypnea/irregular or laboured breathing), tachycardia and hypertension [9,16]. Patients were categorized as cardiorespiratory failure group when presence of respiratory distress with tachycardia, tachypnea, rales, and pink frothy secretion, with bilateral pulmonary infiltrates on chest radiography [9,16].

Clinical samples, including rectal swab or stool sample, were collected during hospitalization. Samples were tested for EV-71, CV-A16 and Human Enterovirus using fluorescent real-time RT–PCR with commercially available kits (Beijing Kinghawk or Jiangsu Shuoshi, China) by the local Centers for Disease Control (CDC) between January 2010 and December 2015, as described previously by Henan CDC [17]. Between May 2014 and December 2016, a third kit (Jiangsu Mole, China) was introduced at the hospital laboratory and used for direct clinical care. If the results tested by CDC and the hospital were inconsistent, virological data were based on the virological result by CDC, with additional details in the supplementary (Supplementary Fig. S1). These test kits were approved by the State Food and Drug Administration of China and have high specificity. Both these test results were used to identify patients for inclusion in our study.

### Outcome measurements

Children were invited to the hospital from March 2017 to May 2019 for neurologic examination, neurodevelopmental assessment and additional investigations, including MRI, lung function tests, and electromyography as clinically appropriate. Type of residence, education level of the parents, medical history and rehabilitation undertaken between discharge and follow-up were collected by interviewing parents or guardians.

#### Neurologic examination

Pediatric rehabilitation neurologists performed a detailed neurological examination in the outpatient clinic, and recorded abnormal findings on a structured case record form. This examination recorded: cranial nerve and ocular abnormalities (e.g. nystagmus, facial palsy, oculomotor palsy, bulbar paralysis (dysphagia and dysarthria)), hearing impairment (reported by parents), gait, ataxia, muscle weakness or amyotrophy, superficial reflexes, deep tendon reflexes, plantar reflexes, urinary or fecal incontinence, seizures and any other neurological abnormalities noted during the course of the examination. Neurologic examination with any abnormal finding was defined as abnormal, irrespective of severity.

#### Neurodevelopmental assessment

Age-appropriate standardized instruments validated in mainland China were administered to the child by trained professional examiners. For assessment of cognitive and language development, we applied the Wechsler Preschool and Primary Scale of Intelligence, Fourth edition (WPPSI-IV) for children aged 3–6 years, and the Wechsler Intelligence Scale for Children, Fourth edition (WISC-IV) for children aged over 6 years [18,19]. For assessment of motor function, we used the Movement Assessment Battery for children-2 (MABC-2) for children aged beyond 3 years, which measures movement ability in three subtests: manual dexterity, aiming and catching, and balance [20]. Parent or primary caregiver administered Adaptive Behavior Assessment System, Second Edition (ABAS-II) questionnaire to assess adaptive skills of daily living [21].

According to recognized criteria [8,22–24], the results of these assessments were classified as good or poor. Lower scores indicate greater impairment in all assessments. The results were considered poor in cognitive, language, motor and adaptive skills domains separately, when WPPSI-IV and WISC-IV Full-scale Intelligence quotient (FSIQ) <85; WPPSI-IV and WISC-IV verbal comprehension index (VCI) <85; MABC-2 total scaled score ≤7; and ABAS-II general adaptive score < 85.

#### Additional investigations

Patients with abnormal MRI results during acute episode were followed up with brain and/or spine MRI examinations accordingly. Any patients with abnormal MRI during acute episode who had subsequently had a normal MRI during routine clinical follow-up did not undergo MRI as part of the study, but were included in the MRI analysis. T1-weighted (T1WI), T2-weighted (T2WI), fluid attenuated inversion recovery (FLAIR) and diffusion-weighted sequences were acquired. The MRI images were reviewed by two radiologists at the time of the examination for clinical care. Scans were classified as normal or abnormal and the lesions detected were summarized.

Lung function tests for children over 6 years and tidal breathing test for children under 6 years were performed at follow-up in those who received mechanical ventilation during hospitalization. Lung ventilation indices included max vital capacity (VCmax), forced vital capacity (FVC), forced expiratory volume in one second (FEV1), FEV1/FVC ratio, maximum mid-expiratory flow (MMEF), forced expiratory flow at 50% vital capacity (FEF50) and forced expiratory flow at 75% vital capacity (FEF75). Tidal breathing indices included time to peak tidal expiratory flow as proportion of expiratory time (TPTEF/TE), tidal volume per kilogram (VT/kg). According to the guideline of pediatric lung function [25–27], we classified lung function as normal; obstructive (FEV1/FVC <92% of the predicated values, or TPTEF/TE <28%), in addition, small airway obstruction (normal FEV1 and normal FEV1/FVC and two of three indexes (MMEF, FEF50, FEF75) below 65% of the predicated values) indicated early stage of obstructive ventilation dysfunction, which is included in obstructive; restrictive (VCmax <80% and normal FEV1/FVC, or VT/Kg <6 ml/kg and normal TPTEF/TE); mixed (obstructive and restrictive). Electromyography (EMG) was performed on patients with neurological deficits at follow-up.

### Statistical analysis

The characteristics of patients are described as frequencies and percentages or median and interquartile range (IQR), including demographics, clinical course, and treatment. Neurodevelopment outcomes, MRI findings, and lung function are described as numbers and percentage. Categorical variables between different complications were compared using χ^2^ test or Fisher exact test as appropriate. The Kruskal–Wallis test and Mann–Whitney U test was used for non-normally distributed continuous variables. Analysis of variance and t-tests were used for normally distributed continuous variables. We used logistic regression analyses to explore the risk factors for poor neurodevelopmental outcomes in cognitive, motor, language and adaptive domains separately. Age at onset, sex, residence type, highest educational level of parents, follow-up period and clinical complication, which may be related to long-term neurological sequalae were included in the univariate logistic regression. Variables with *P* value < 0.1 in univariate analysis were included in all multivariate logistic regression. Two-sided α < 0.05 was considered statistically significant. Bonferroni correction was used to adjust the *P* value for multiple comparison. All analyses were performed in R version 3.6.1.

### Ethics

The study was approved by the Institutional Review Boards of Henan Children’s Hospital (IRB#YZ-17-005) and Public Health School of Fudan University (IRB#2017-12-0652). Written informed consent was obtained from parents or guardians of all study participants.

## Results

### Study population

**Figure 1.**
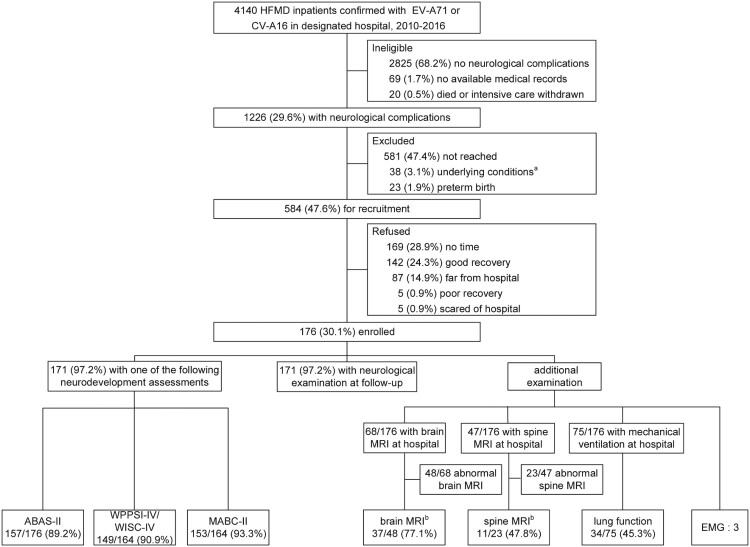
Flow chart for the enrolment and the assessments performed at follow-up of 176 patients. ^a^: 38 patients with underlying conditions: 16 with prior learning disability or neurological regression, 6 with prior chronic respiratory or cardiac illness, 13 with previous pediatric intensive care unit admission and 3 with prior delayed development. ^b^: MRI results included available reexamination results after the acute episode to follow-up (8 brain MRI and 2 spine MRI) by reviewing picture archiving and communication system. Numbers of patients applied in the neurodevelopment assessments may differ due to different age covered by the different assessments. *ABAS-II* Adaptive Behavior Assessment System, second Edition. *WPPSI-IV* Wechsler preschool and primary scale of intelligence, Fourth edition. *WISC-IV* Wechsler intelligence scale for children, fourth edition. *MABC-II* Movement Assessment Battery, second edition. *MRI* Magnetic Resonance Imaging. *EMG* Electromyography.

We identified 1226 patients with CNS involvement among 4140 HFMD patients with EV-A71 or CV-A16 infection. Of those, 584 patients met the criteria for case entry and 176 (30.1%) were enrolled for follow-up (Figure 1). The total number of laboratory-confirmed cases, neurological cases and participants were plotted by onset date (Supplement Fig. S1). There was no significant difference in the age at onset or sex between participants and those who either refused or were not available for any reason, but participants had longer hospital stay (Supplement Table 1).

Of the 176 participants, 24 (13.6%) suffered CNS involvement, 133 (75.6%) ANS dysregulation and 19 (10.8%) cardiorespiratory failure. Encephalitis, brainstem encephalitis and encephalomyelitis accounted for 0 (0%), 22 (92%) and 2 (8%) cases respectively in CNS groups, and 4 (3%), 116 (87%) and 13 (10%) respectively in those with ANS dysfunction. Five patients had CV-A16 infection, including two cases of brainstem encephalitis, one encephalomyelitis and two ANS dysregulation; one patient had EV-A71 and CV-A16 dual-infection with ANS dysregulation; the remainder had EV-A71 infection. A total of 62% participants were younger than two years at the onset of HFMD and 66% were male. The median follow-up period was 4.4 years (IQR 1.9-6.1, range 1.4-8.3) and 43% (75 of 176) patients treated with mechanical ventilation (Table 1).

**Table 1.** Demographics, clinical courses and treatment characteristics by complications.

Characteristics	Overall (*n *= 176)	CNS involvement (*n *= 24)	ANS dysregulation (*n *= 133)	Cardiorespiratory failure (*n *= 19)	*P* value
Demographics					
Age at onset, y, median [IQR]	1.7 [1.3, 2.5]	1.9 [1.3, 2.6]	1.8 [1.3, 2.6]	1.4 [1.0, 1.7]	0.012
Age group at onset, No. (%)					
0∼1 y	109 (62)	13 (54)	80 (60)	16 (84)	0.091
2–9 y	67 (38)	11 (46)	53 (40)	3 (16)	
Male, No. (%)	116 (66)	17 (71)	85 (64)	14 (74)	0.604
Rural residence, No. (%)	77 (44)	11 (46)	56 (42)	10 (53)	0.671
Parents education, No. (%)					
High school or below	112 (64)	12 (50)	87 (65)	13 (68)	0.317
Junior college or above	64 (36)	12 (50)	46 (34)	6 (32)	
Age at follow-up, y, median [IQR]	6.4 [4.4, 8.0]	4.7 [3.4, 7.6]	6.6 [4.6, 8.0]	7.1 [4.8, 7.9]	0.207
Follow-up period, y, median [IQR]	4.4 [1.9, 6.1]	1.9 [1.8, 4.5]	4.4 [2.0, 6.1]	4.6 [3.3, 6.7]	0.027
Course of disease					
Hospitalization days, median [IQR]	11.0 [8.0, 15.0]	8.0 [5.8, 9.2]	11.0 [8.0, 15.0]	18.0 [16.0, 26.5]	<0.001
ICU admission, No. (%)	155 (88)	10 (42)	126 (95)	19 (100)	<0.001
ICU duration, days, median [IQR]	7.0 [4.0, 11.0]	4.0 [4.0, 5.0]	7.0 [4.0, 10.0]	15.0 [9.5, 24.5]	<0.001
Treatment					
Mechanical Ventilation, No. (%)	75 (43)	0 (0)	56 (42)	19 (100)	<0.001
Duration of mechanical ventilation, days, median [IQR]	4.0 [4.0, 6.0]	-	4.0 [4.0, 5.0]	7.0 [5.0, 15.0]	<0.001
IVIG, No. (%)	154 (88)	15 (62)	121 (91)	18 (95)	0.001
Corticosteroid, No. (%)	175 (99)	24 (100)	132 (99)	19 (100)	1

Note: IQR: interquartile range. IVIG: intravenous immunoglobulin.

### Outcome data

The overall number of children completed each exanimation during follow-up is shown in Figure 1. A long duration of testing time, or that parents or primary caregivers did not perceive the tests to be helpful, were the most common reasons for not completing examinations at follow-up. The number of patients who could not be successfully tested due to the children’s noncompliance were 2, 5 and 5 in neurological examination, WPPSI-IV/WISC-IV and MABC-II assessments, respectively.

**Figure 2.**
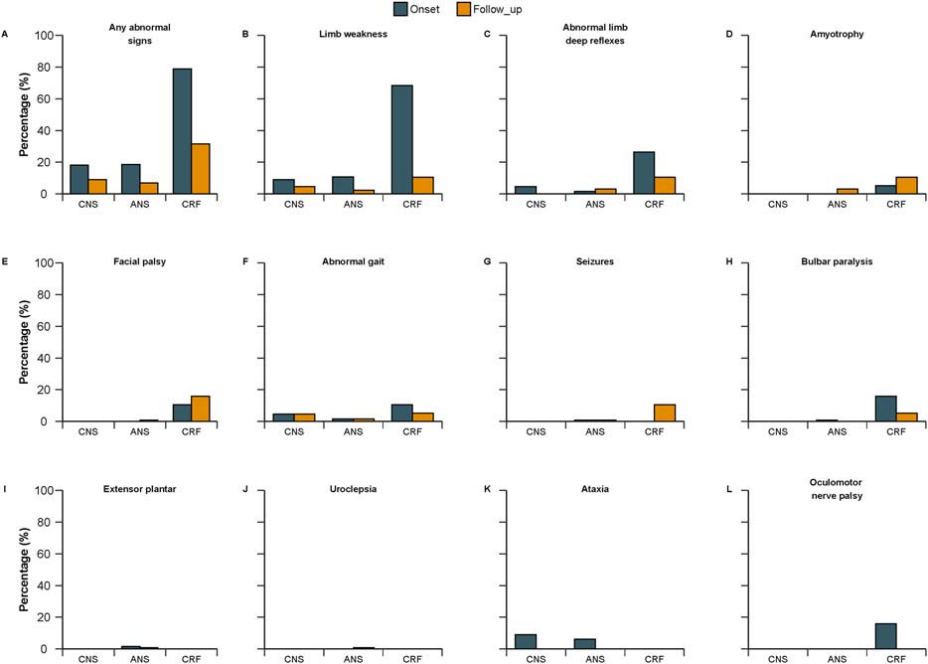
Detailed neurologic examination outcome at discharge and at follow-up by complications of 171 patients. Five cases refused neurological examination at follow-up. Each panel represents a neurologic finding except panel A is overall. The panel ranks neurologic examination outcome in decreasing order of overall percentage of outcome at follow-up. CNS central nervous system. ANS autonomic nervous system dysregulation. CRF cardiorespiratory failure.

Neurological abnormalities were observed in 25% of patients (43 of 171) at discharge, and in 10% (17 of 171) of patients at follow-up. The abnormalities observed were muscle weakness (6, 3.5%), abnormal limb deep reflexes (6, 3.5%), amyotrophy (6, 3.5%), facial palsy (4, 2.3%), abnormal gait (4, 2.3%), seizures (3, 1.8%), bulbar paralysis (dysarthria, 1, 0.6%), unilateral extensor plantar (1, 0.6%) and urinary incontinence when nervous (1, 0.6%) (Figure 2). Muscle weakness was the major defect at both discharge and follow-up. Three cases with EMG showed peripheral nerve lesions, indicating peripheral neuropathy. None had superficial reflex, dysphagia, hearing or fecal incontinence problems. None of our participants required tube feeding or ventilation at follow-up. One patient with dyspnea required supplementary oxygen at home for about 5 months after discharge. This patient’s illness was complicated by recurrent pneumonia and the patient underwent plication of the diaphragm 11 months after illness onset due to right diaphragmatic eventration. The phrenic nerves were found to be unresponsive to stimulation during surgery. An example of sequelae following severe HFMD is shown in Supplement Fig. S2.

The rates of poor outcome (Table 2) were significantly different between CNS, ANS and cardiorespiratory complications in motor (28%, 38%, 71%) domain (p=0.020), but not for cognitive (20%, 24%, 35%), language (25%, 36%, 41%) and adaptive (24%, 16%, 26%) domains (*p* = 0.537, *p* = 0.551, *p* = 0.403). Comparison of the scores obtained for each neurodevelopment assessment showed a similar pattern with the CNS group consistently scoring more highly than the other groups (Supplement Fig. S3).

**Table 2.** Outcomes of neurodevelopment assessment, lung function tests and MRI by complications.

Variables	Overall (n = 176)	CNS involvement (n = 24)	ANS dysregulation (n = 133)	Cardiorespiratory failure (n = 19)	*P* value
Poor outcomes of neurodevelopment assessment					
Cognitive development	37/149 (25)	4/20 (20)	27/112 (24)	6/17 (35)	0.537
Motor function	62/153 (41)	5/18 (28)	45/118 (38)	12/17 (71)	0.020
Language development	52/149 (35)	5/20 (25)	40/112 (36)	7/17 (41)	0.551
Adaptive skills	29/157 (18)	5/21 (24)	19/117 (16)	5/19 (26)	0.403
Lung function tests					
No. of patients	34	0	23	11	
Total ventilation dysfunction	15/34 (44)	-	9/23 (39)	6/11 (55)	0.475
Obstructive	14/34 (41)	-	9/23 (39) ^a^	5/11 (45) ^b^	1
Restrictive	0/34 (0)	-	0/23 (0)	0/11 (0)	-
Mixed	1/34 (3)	-	0/23 (0)	1/11 (9)	0.324
Brain MRI scans					
No. of patients	37	7	23	7	
Persistent abnormalizes	6/37 (16)	0/7 (0)	2/23 (9)	4/7 (57)	0.014
Resolved ^c^	31/37 (84)	7/7 (100)	21/23 (91)	3/7 (43)	
Spine MRI scans					
No. of patients	11	3	7	1	
Persistent abnormalizes	0/11 (0)	0/3 (0)	0/7 (0)	0/1 (0)	-
Resolved ^a^	11/11 (100)	3/3 (100)	7/7 (100)	1/1 (100)	

Note: Data are presented numbers/numbers (percentages). MRI: magnetic resonance imaging.

^a^ There were two children with small airway obstruction among nine children with obstructive ventilation dysfunction.

^b^ There were one child with small airway obstruction among five children with obstructive ventilation dysfunction.

^c^ Resolved results of MRI scan include available normal reexamination results after the acute episode to follow-up by reviewing picture archiving and communication system (8 brain MRI and 2 spine MRI).

Lung function tests were performed on 34 patients whom had been ventilated during hospitalization. The cardiorespiratory failure group had more patients with an obstructive picture on lung function testing than the ANS group, though this difference did not achieve statistical significance (6/11 (55%) vs. 9/23 (39%), *p* = 0.475, Table 2). In the ANS group, six mild and one moderate obstructive, and two small airway obstructive pictures were identified. In the cardiorespiratory failure group, two mild, one moderate and one severe obstruction, one small airway obstructive and one severe mixed obstructive/restrictive picture with significant dyspnea were identified. Otherwise, the children did not spontaneously report respiratory symptoms, and were able to keep up with their peers in most settings. However, the parents reported subjective breathlessness in their children when exercising.

**Figure 3.**
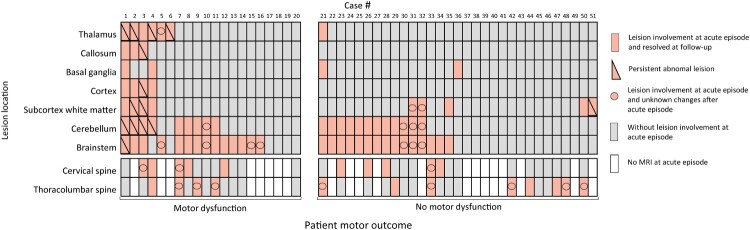
Lesion location identified by MRI for individual case with or without motor dysfunction. Cases were both brain MRI performed at acute episode and motor assessment at follow-up. MRI results included available reexamination results after the acute episode to follow-up (5 brain MRI and 2 spine MRI) by reviewing picture archiving and communication system. Motor dysfunction is defined total motor scaled score ≤7 of MABC-2 evaluation. The number is case ID. Thalamus and cortex has significant difference between motor dysfunction and no motor dysfunction at follow-up (*p* = 0.011, *p* = 0.019).

A total of 68 cases had brain MRI during acute illness, and 48 cases’ scans were abnormal. At the acute episode, thalamic, corpus callosum, basal ganglia, cortex and subcortex white matter injury were more in cardiorespiratory failure cases whereas cerebellum and brainstem involvement had no statistical significance in various complications (Supplement Table 2). Of those, 37 cases were re-examined by brain MRI and 6 patients (16%) had persistent abnormalities, mainly thalamic and/or cerebellar lesions (Table 2). A total of 47 cases had spine MRI at acute stage and 11 cases with abnormal results were reexamined by MRI. No abnormalities were detected on spinal MRI in all groups (Table 2). Thalamic and cortical involvement in the acute stage were significantly associated with poor motor outcome (30% (6 of 20) vs. 3% (1 of 31) *p* = 0.01, 20% (4 of 20) vs. 0% (0 of 31) *p* = 0.02) (Figure 3). The thalamic changes were present on FLAIR and T2WI in all cases, all of these patients had been ventilated. Subcortical white matter involvement in the acute stage was significantly associated with poor long-term language outcome (43% (6 of 14) vs. 11% (4 of 36) *p* = 0.02).

## Discussion

Our study systematically and comprehensively assessed the type and rate of long-term sequelae of HFMD caused by EV-A71 or CV-A16 infection following the emergence in mainland China. We report similar neurological findings to Chang et al. and Teoh et al., particularly focal limb weakness and atrophy [8,9]. The study included inpatients between 1998 and 2003 found 40% (32 of 81) patients with severe CNS involvement (including encephalitis, a poliomyelitis-like syndrome, and encephalomyelitis) with or without cardiorespiratory failure had neurological sequalae, compared with 10% (17 of 171) in our study [8]. In the intervening time between the previous study and ours, a series of guidelines for management of HFMD was released and improved by the Ministry of Health of the People’s Republic of China and by WHO [16,28]. This would be expected to improve both the acute in-patient management and the rehabilitation of these cases, and likely accounts for the reduced rate of neurological sequelae in our study. In another study conducted in Australia contemporaneously closer to this study in 2013, 8.8% of patients (5 of 57) had focal paresis 12 months after the acute stage [9], a similar rate to this study.

The cardiorespiratory failure group suffered the worst outcome in all domains, suggesting that these patients were the most severely affected following HFMD, consistent with other studies [8,9]. The patients with ANS seemed to have poorer outcome in cognition, motor and language domains than patients with CNS only but allowing for multiple comparisons, this difference was not statistically significant. The ANS stage can be considered to be equal to early cardiopulmonary failure [16,29]. Therefore, management of ANS dysregulation in children is important to prevent progression to cardiopulmonary failure and improve long-term outcomes [30].

The language problem related to HFMD has not been emphasized before. Enterovirus are neurotropic and may cause speech and language impairments by induce CNS cell apoptosis [10]. Language problems are often not associated with marked structural abnormalities in the brain using currently available imaging techniques, although a small proportion of children were identified with abnormalities such as white matter or language cortex lesions [31]. Parents should pay more attention to the children’s language development not only academic attainment.

We presented data on long-term MRI changes compared with other short-term studies. Most of the abnormalities on brain MRI, and all of those on spine MRI, resolved over time in our study, which indicates the damage is likely not progressive. Consisting with the study followed for six months to one year in Guangzhou [13], patients with thalamic injury in the acute stage had poor motor prognosis, whereas persistent thalamic lesions at follow-up confirmed the effects on motor function. The thalamus plays a key role in connecting specific pathways from the basal ganglia and cerebellum to the motor cortex [32]. Our study did not observe brainstem changes affected long-term motor function although this has been observed in other studies [9,13]. Case definitions of clinical severity and measurement of outcome, especially the length of the follow-up period, may influence this relationship.

Lung function of HFMD patients who have been mechanically ventilated in the acute stage has not previously been studied. All of the abnormalities detected on lung function testing were obstructive in nature, indicating chronic airflow limitation, except for one patient with scoliosis who showed a mixed obstructive/restrictive picture, and was subjectively more severely affected. There are multiple interpretations of this obstructive defect. The first is that prolonged mechanical ventilation causes respiratory muscle atrophy with structural change [33]. Secondly immunological changes and weakened lung barriers after enterovirus infection increased the risk of allergic diseases such as asthma [10,34,35]. The mixed obstructive/restrictive dysfunction was caused by unilateral phrenic nerve palsy and scoliosis. We presume that phrenic nerve palsy due to involvement of the 2nd to 6th cervical nerve roots rather than congenital phrenic dysplasia, lead to diaphragmatic eventration [36]. Respiratory function improved but did not recover completely after diaphragm plication surgery. We report here the first case of scoliosis caused by HFMD. The anterior horn cell damage in this case is likely not progressive, but the result of the damage is scoliosis as the child grows. Understanding the different types of ventilatory dysfunction will guide treatment to improve respiratory function.

Motor function is most sensitive to impairment. In our study, motor function refers to developmental status of fundamental movement skills [37]. Motor development problems can be explained by motor neuron damage during acute disease causing limb weakness/atrophy and subsequent functional effects [38]. EMG at follow-up also showed peripheral nerve lesions. But three participants had normal motor development scores after experiencing early and effective rehabilitation, although they still had unilateral limb weakness/atrophy at follow-up. This indicates that early intervention can be beneficial for children to reach age-appropriate movement skills. Additionally, some patients without limb weakness/atrophy had abnormal motor development scores. This may, in part, be accounted for by abnormalities in motor function secondary to attention deficits or intellectual disabilities caused motor coordination disorder. There has been more work on attention-deficit hyperactivity disorder (ADHD) following enterovirus infections, and children with EV-A71 infection are more likely to be inattentive and hyperactivity-impulsivity [39,40]. In population-based studies, ADHD was reported to occur alongside developmental coordination disorder (DCD) in 50% of cases, and vice versa [24]. Medical staff and parents caring for children with EV-A71 infection should be alert to the presence of ADHD and DCD. Further work should be aimed at uncovering common mechanisms of ADHD and DCD in children with EV-A71 infection.

We also identified adaptive problems in our cohort although the comparison in abnormal adaptive rates between groups was not significant. There is generally a correlation between intellectual functioning and adaptive behaviour and the grand mean of the correlations in IQ groups of ≥90 in participants aged 5–12 is about 0.4 [41]. In our study the Pearson correlation coefficient for FSIQ and adaptive behaviour was poor, at 0.31, but similar to the literature average. The cardiorespiratory failure group had a higher rate of abnormalities in the society and practical subscales of adaptive behaviour scales than other two groups. Adaptive problems reflect social issue, such as communication and independent living among children affected by severe HFMD. In our univariate and multivariate analysis for factors that predict poor outcome (Supplement Table 3, Table 4), illness severity during the acute stage predicted poor motor outcome, whereas the educational attainment of parents was protective in cognitive, language and adaptive outcomes. The presence of appropriate supports from caregivers can improve individual life functioning [42].

There was one patient with ANS dysregulation with CV-A16 infection had left upper limb weakness and amyotrophy. Outcomes following severe HFMD to CV-A16 infection were less severe than EV-A71 infection but still suffered from poor prognosis. The effectiveness of EV-A71 vaccination in the real-word is estimated to be 85.4%, but it didn’t have cross immunity effect for preventing CV-A16 and CV-A6 [43]. Studies of the outcomes of severe HFMD to other enterovirus serotypes (e.g. CV-A6, CV-A4, etc.) are required in the future, which might become the dominant circulating virus after license of EV-A71 vaccine.

Our study design reduced recall bias because the clinical data were acquired from medical records, whereas outcome indicators were measured at follow-up. Nevertheless, our study has limitations. The first was the high rate of patients’ families who could not be reached or who refused to participate in our study. There may be a tendency for more serious cases to attend follow-up to seek healthcare assistance, whereas this might be perceived as futile by those who have recovered well. The second limitation is that we assumed that the neurological examination at discharge was normal if symptoms or signs were not reported and recorded as notable. The third limitation is that the diagnosis of ANS dysregulation is based on the clinical features, as per WHO, and we did not evaluate the autonomic nervous system during follow-up. A fuller assessment of autonomic function at follow up remains an important outstanding topic for future research. The differences in cognitive, language and adaptive domains between groups were not significant, possibly due to sample sizes. Finally, our study was performed in a single centre, possibly limiting generalizability. Multicentre studies could be conducted to fully understand the long-term burden of HFMD.

## Conclusion

In conclusion, we have observed that HFMD with neurologic complications may have abnormalities in neurological, motor, language, cognition domain and adaptive and ventilatory function. Our study provides a comprehensive dataset, and indicates the value of interventions. Our data strongly suggest that improvements in acute management in China have resulted in better outcomes for patients. Early and targeted interventions are meaningful for treatment in the clinical acute stage and ensuring the best outcome for subsequent childhood development. EV-A71 vaccination is essential and should be promoted to reduce disease burden.

## Supplementary Material

Supplemental Material
