# Supplementary material for: Long-term neurodevelopment outcomes of hand, foot and mouth disease inpatients infected with EV-A71 or CV-A16, a retrospective cohort study
Source: Emerg Microbes Infect. 2021 Mar 29;10(1):545–54. doi: 10.1080/22221751.2021.1901612 (PMC8009121; doi:10.1080/22221751.2021.1901612)
Supplement: Supplemental Material [file TEMI_A_1901612_SM6950.docx]

**Supplementary**

**Supplementary Table S1.** Demographics comparison for participants and those that not enrolled into follow-up.

**Supplementary Table S2.** MRI of the brain and/or spine at acute episode in HFMD cases by complication.

**Supplementary Table S3.** Univariate logistic regression analysis results predicting poor outcome.

**Supplementary Table S4.** Multivariate logistic regression analysis results predicting poor outcome.

**Supplementary Fig. S1.** Reported patients hospitalized with EV-A7 or CV-A16 virus infections in Henan Children’s Hospital between 2010 and 2016

**Supplementary Fig. S2.** A 12-month-old patient (onset age) with poor prognosis at follow-up.

**Supplementary Fig. S3.** Individual scores in the neurodevelopment domains by complications.

**Supplementary Table 1. Demographics comparison for participants and those that not enrolled into follow-up**

| **Characteristics** | **Recruitment**  **n= 584** | | | **With neurologic complications**  **n= 1226** | | | **Confirmed laboratory infection**  **n= 4051** | | |
| --- | --- | --- | --- | --- | --- | --- | --- | --- | --- |
|  | **Enrolled**  **n=176** | **Refused**  **n=408** | ***P* value** | **Recruited**  **n=584** | **Not available**  **n=642** | ***P* value** | **With neurologic complications**  **n=1226** | **Without neurologic complications**  **n=2825** | ***P* value** |
| Age at onset, years, median [IQR]) | 1.7 [1.3, 2.5] | 1.7 [1.3, 2.5] | 0.736 | 1.7 [1.3, 2.5] | 1.7 [1.2, 2.5] | 0.349 | 1.7 [1.2, 2.5] | 1.8 [1.3, 2.7] | 0.019 |
| Sex (male, n (%)) | 116 (66) | 266 (65) | 0.943 | 382 (65) | 424 (66) | 0.863 | 806 (66) | 1766 (63) | 0.054 |
| Hospitalization days, median [IQR] | 11 [8, 15] | 10 [7, 13] | 0.012 | 10 [8, 13] | 10 [8, 14] | 0.687 | 10 [8, 13] | 5 [4, 6] | <0.001 |

Comparison for patients with neurologic complications between those who were enrolled into the follow up study and those who refused, and between those who were recruited and those who were not available for any reason; and comparison for patients with EV-A71 or CV-A16 infection between those who were with neurologic complications and those who were without neurologic complications.

**Supplementary Table 2.** **MRI of the brain and/or spine at acute episode in HFMD cases by complication.**

| **Variables** | **Overall**  **(n=176)** | **CNS**  **involvement**  **(n=24)** | **ANS**  **dysregulation**  **(n=133)** | **Cardiorespiratory failure**  **(n=19)** | **P value** |
| --- | --- | --- | --- | --- | --- |
| **Number of cases with brain MRI** | 68 | 15 | 44 | 9 |  |
| Thalamus | 8 (12) | 0 (0) | 2 (5) | 6 (67) | <0.001 |
| Callosum | 3 (4) | 0 (0) | 0 (0) | 3 (33) | 0.002 |
| Basal ganglia | 4 (6) | 0 (0) | 1 (2) | 3 (33) | 0.008 |
| Cortex | 4 (6) | 0 (0) | 0 (0) | 4 (44) | <0.001 |
| Subcortex white matter | 11 (16) | 3 (20) | 3 (7) | 5 (56) | 0.002 |
| Cerebellum | 30 (44) | 7 (47) | 18 (41) | 5 (56) | 0.704 |
| Brainstem | 42 (62) | 8 (53) | 26 (59) | 8 (89) | 0.184 |
| **Number of cases with spine MRI** | 47 | 12 | 29 | 6 |  |
| Cervical spine | 12 (26) | 3 (25) | 7 (24) | 2 (33) | 0.892 |
| Thoracolumbar spine | 15 (32) | 5 (42) | 8 (28) | 2 (33) | 0.734 |

Data are presented numbers/numbers (percentages).

**Supplementary Table 3.** **Uni****variate logistic regression analysis results predicting poor outcome**

| **Variables** | **Cognitive outcome** | | **Motor outcome** | | **Language outcome** | | **Adaptive outcome** | |
| --- | --- | --- | --- | --- | --- | --- | --- | --- |
|  | **OR (95%CI)** | **P value** | **OR (95%CI)** | **P value** | **OR (95%CI)** | **P value** | **OR (95%CI)** | **P value** |
| Age at onset |  |  |  |  |  |  |  |  |
| ≥2 years | Ref | - | Ref | - | Ref | - | Ref | - |
| 0-1 years | 2.43 (1.10-5.70) | 0.033 | 1.33 (0.69-2.60) | 0.398 | 1.00 (0.51-1.99) | 0.996 | 0.85 (0.38-1.97) | 0.698 |
| Sex |  |  |  |  |  |  |  |  |
| Female | Ref | - | Ref | - | Ref | - | Ref | - |
| Male | 0.95 (0.44-2.08) | 0.893 | 0.73 (0.37-1.43) | 0.353 | 1.16 (0.58-2.38) | 0.671 | 1.16 (0.5-2.89) | 0.731 |
| Residence |  |  |  |  |  |  |  |  |
| Urban | Ref | - | Ref | - | Ref | - | Ref | - |
| Rural | 2.75 (1.29-5.98) | 0.009 | 0.94 (0.49-1.81) | 0.859 | 2.00 (1.01-4.00) | 0.047 | 2.96 (1.30-7.13) | 0.012 |
| Highest educational level of parents |  |  |  |  |  |  |  |  |
| High school or below | Ref | - | Ref | - | Ref | - | Ref | - |
| Junior College or above | 0.22 (0.07-0.55) | 0.003 | 1.28 (0.65-2.52) | 0.466 | 0.31 (0.13-0.67) | 0.004 | 0.27 (0.09-0.69) | 0.012 |
| Follow-up period (years) | 0.87 (0.71-1.05) | 0.145 | 1.08 (0.91-1.28) | 0.396 | 1.11 (0.93-1.33) | 0.245 | 0.99 (0.81-1.22) | 0.956 |
| Complications |  |  |  |  |  |  |  |  |
| CNS | Ref | - | Ref | - | Ref | - | Ref | - |
| ANS | 1.27 (0.42-4.72) | 0.690 | 1.60 (0.56-5.27) | 0.399 | 1.67 (0.60-5.43) | 0.355 | 0.62 (0.21-2.08) | 0.403 |
| Cardiorespiratory failure | 2.18 (0.51-10.33) | 0.301 | 6.24 (1.53-29.77) | 0.014 | 2.10 (0.52-8.97) | 0.299 | 1.14 (0.27-4.93) | 0.855 |

CNS central nervous system. ANS autonomic nervous system dysregulation.

**Supplementary Table 4. Multivariate logistic regression analysis results predicting poor outcome.**

| **Variables** | **Cognitive outcome** | | **Motor outcome** | | **Language outcome** | | **Adaptive outcome** | |
| --- | --- | --- | --- | --- | --- | --- | --- | --- |
|  | **OR (95%CI)** | **P value** | **OR (95%CI)** | **P value** | **OR (95%CI)** | **P value** | **OR (95%CI)** | **P value** |
| Age at onset |  |  |  |  |  |  |  |  |
| ≥2 years | Ref | - | Ref | - | Ref | - | Ref | - |
| 0-1 years | 2.78 (1.19-6.92) | 0.022 | 1.09 (0.55-2.20) | 0.798 | 1.01 (0.49-2.10) | 0.982 | 0.88 (0.36-2.18) | 0.775 |
| Residence |  |  |  |  |  |  |  |  |
| Urban | Ref | - |  |  | Ref | - | Ref | - |
| Rural | 1.80 (0.79-4.17) | 0.163 | 0.96 (0.46-2.01) | 0.916 | 1.46 (0.69-3.08) | 0.315 | 2.20 (0.91-5.57) | 0.085 |
| Highest educational level of parents |  |  |  |  |  |  |  |  |
| High school or below | Ref | - |  |  | Ref | - | Ref | - |
| Junior College or above | 0.23 (0.07-0.64) | 0.008 | 1.35 (0.64-2.89) | 0.429 | 0.37 (0.15-0.85) | 0.022 | 0.33 (0.1-0.96) | 0.053 |
| Complications |  |  |  |  |  |  |  |  |
| CNS | Ref | - | Ref | - | Ref | - | - | - |
| ANS | 0.82 (0.24-3.21) | 0.752 | 1.64 (0.57-5.43) | 0.382 | 1.43 (0.49-4.84) | 0.529 | 0.49 (0.15-1.76) | 0.247 |
| Cardiorespiratory failure | 1.07 (0.22-5.61) | 0.931 | 6.42 (1.52-31.62) | 0.015 | 1.68 (0.39-7.71) | 0.493 | 0.85 (0.17-4.12) | 0.837 |

CNS central nervous system. ANS autonomic nervous system dysregulation.

**Supplementary Fig. S1.** **Reported patients hospitalized with EV-A7 or CV-A16 virus infections in Henan Children’s Hospital between 2010 and 2016**


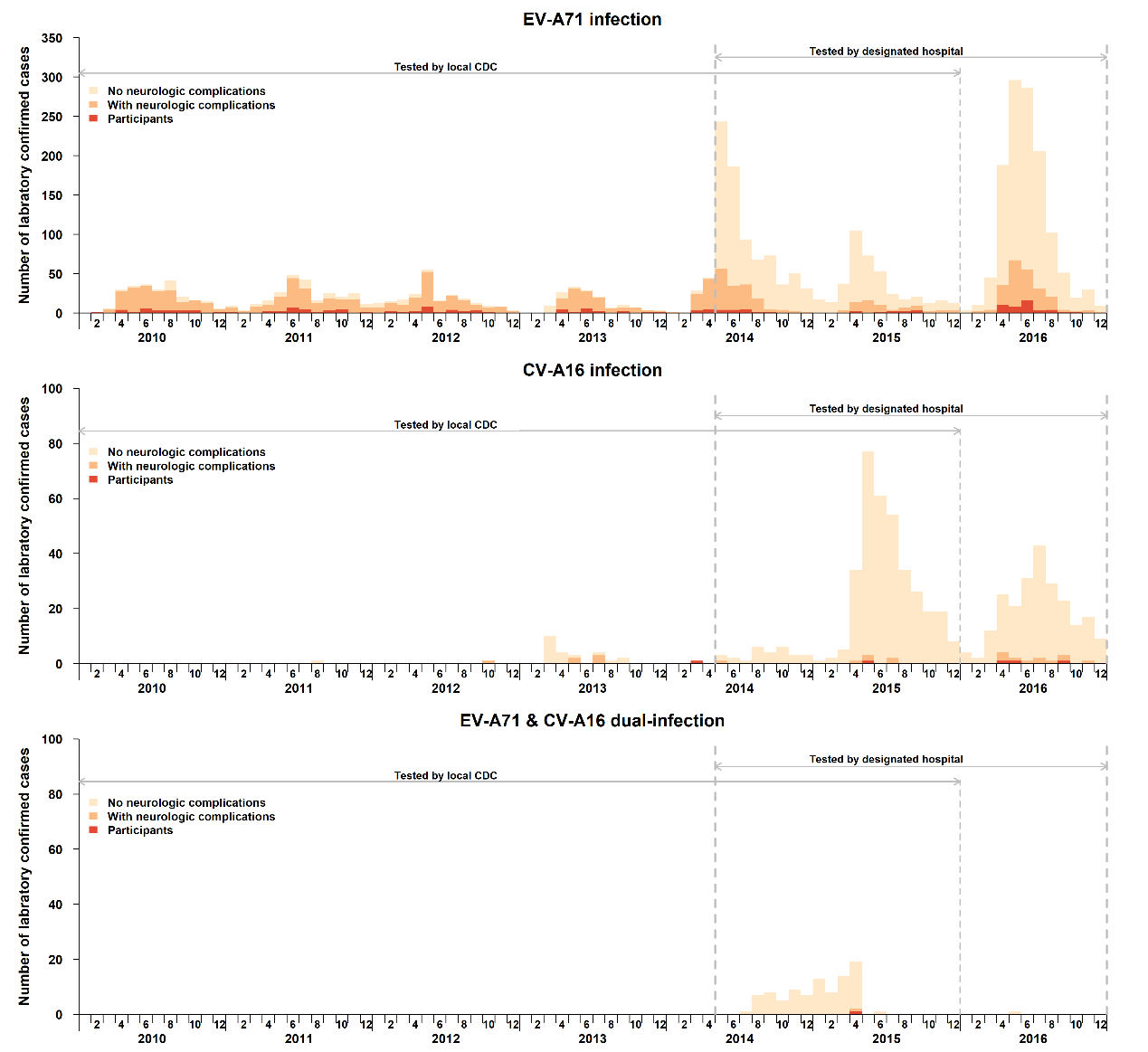


Among 4140 patients, there were 3419, 628 and 93 cases testing positive for EV-A71, CV-A16 and dual-infection (EV-A71 and CV-A16), respectively.

Among 4140 patients, 1190 patients were tested by CDC only, 2873 patients were tested by the hospital only, and 77 patients were tested by both CDC and the hospital. 5 of 77 patients (6.5%) were inconsistent in the results by CDC and the hospital and five patients were based on the virological result by CDC, including four CV-A16 and one EV-A71 (the EV-A71 patient attended the follow-up).

**Supplementary Fig. S2. A 12-month-old patient (onset age) with poor prognosis at follow-up.**


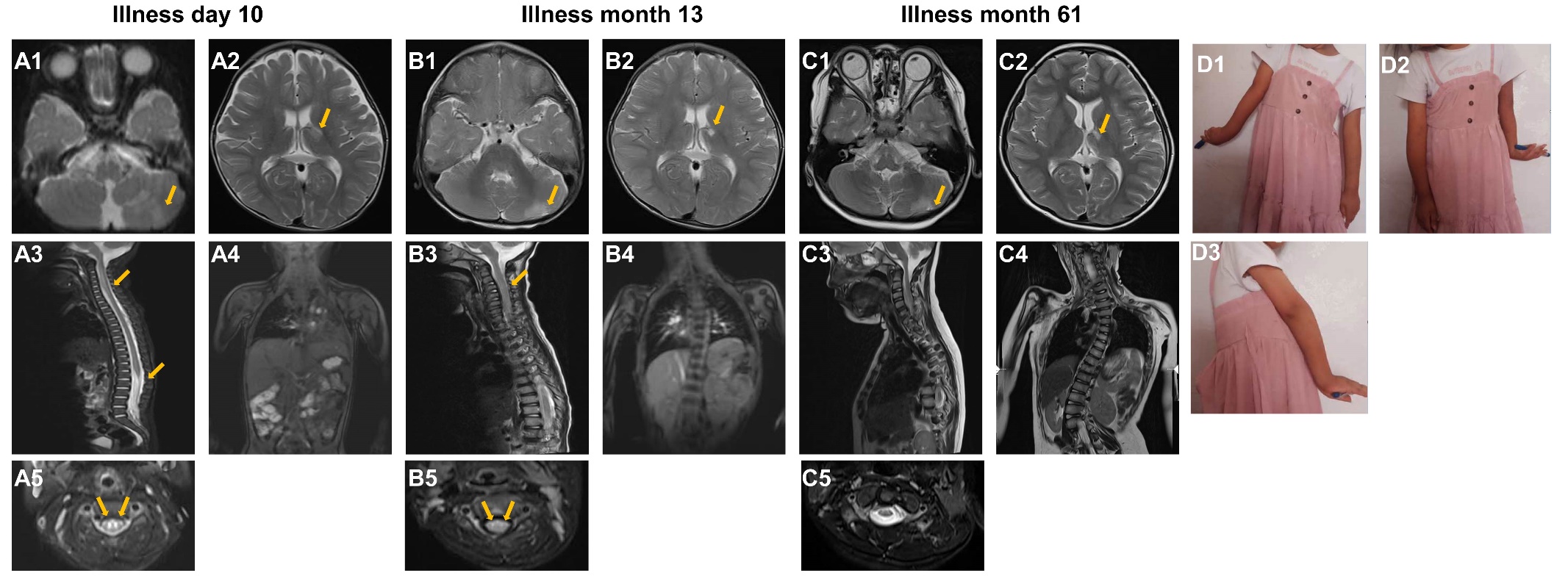


The patient suffered cardiorespiratory failure during the acute illness and had weakness and amyotrophy in residual bilateral upper limbs and scoliosis and received diaphragm plication in illness 11 months due to right diaphragmatic eventration. Shown are MRI scan images obtained on illness day 10 (A1, A2, A3, A4, A5), illness month 13 (B1, B2, B3, B4, B5) and illness month 61 (C1, C2, C3, C4, C5). Arrows indicated the damage.

(A1-A2): high signal areas in left cerebellum, left basal ganglia area, left thalamus and cortex involvement. (B1-B2-C1-C2): improvements compared to A1-A2 after rehabilitation treatments.

(A3): high signal especially in cervical 2- cervical 6 and thoracic 11-12. (B3): improvement of cervical and thoracic session but high signal of cervical 2- cervical 5 still remained compared with A3. (C3): no obvious signal abnormality.

(A3-A4): normal curvature of the spine at onset. (B3-B4-C3-C4): severe scoliosis.

(A5-B5): hyperintense lesions in the anterior horn regions. (C5): no obvious signal abnormality.

The right panel (D1, D2, D3) showing shoulder. Right shoulder flexion 30°, abduction 15-20°; left shoulder flexion 10°, abduction 10°.

**Supplementary Fig. S3. Individual scores in the neurodevelopment** **domains by complications.**


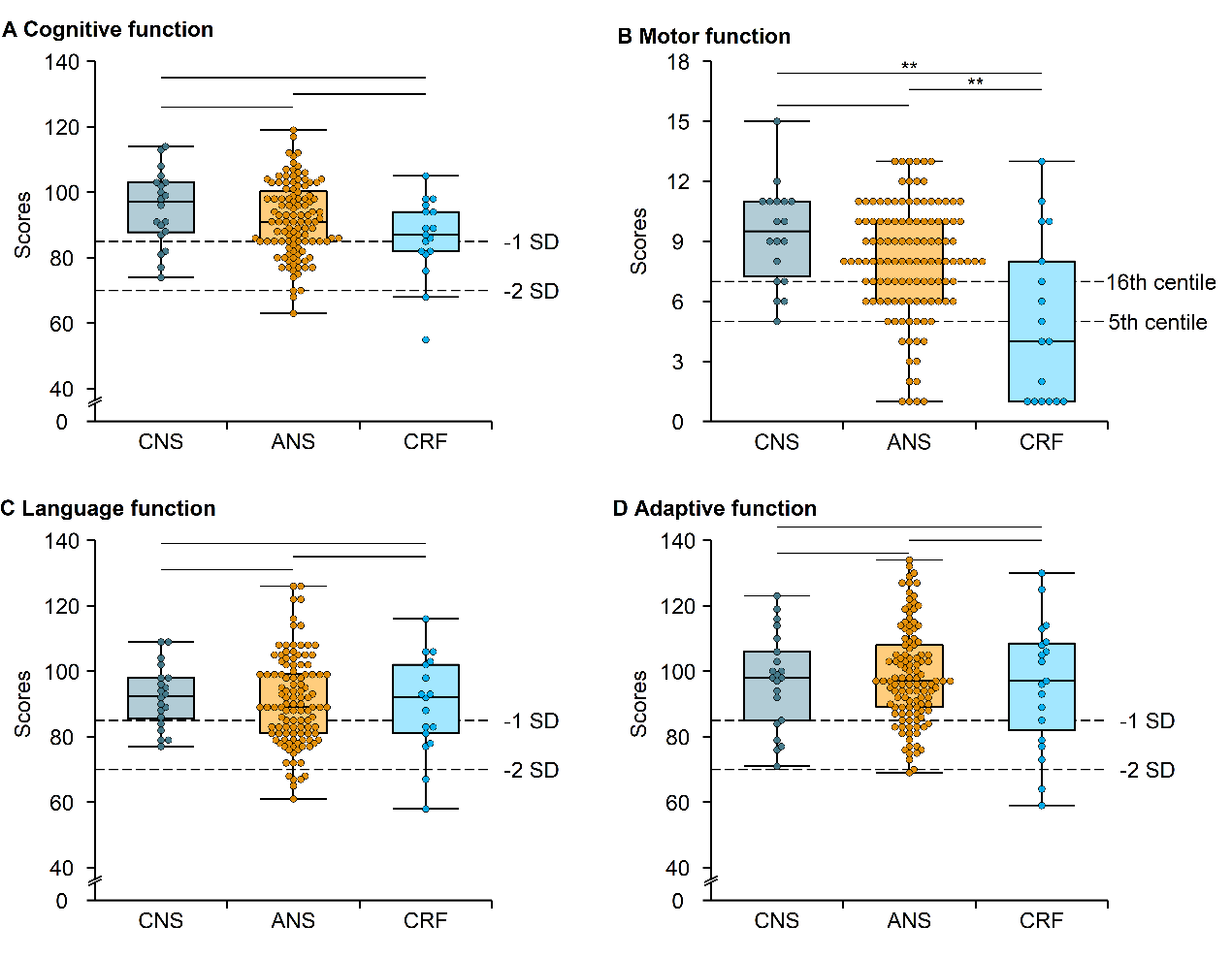


Higher scores show more well neurodevelopment function. Motor domain have significant difference between different complications (p<0.05). Analysis of variance (ANOVA) was used in cognitive, language and adaptive domain except Kruskal-Wallis test was used in motor domain because motor score was non-normally distributed. Box plot shows median, upper and lower quartiles, 1.5 *inter-quartile ranges from upper and lower quartiles. The horizontal lines at the top of each panel refer to multiple comparison. Bonferroni correction were used for multiple comparison. Symbol *** indicate p<0.001, ** indicate p<0.01 and * indicate p<0.05. *CNS* central nervous system. *ANS* autonomic nervous system dysregulation. *CRF* cardiorespiratory failure.

**A:** **Cognitive function:** Full Scale intelligence quotient (FSIQ) scores on the Wechsler Preschool and Primary Scale of Intelligence, Fourth edition (WPPSI-IV) and Wechsler Intelligence Scale for Children, Fourth edition (WISC- IV).

**B: Motor function:** Motor total scaled scores on the Movement Assessment Battery for children-2 (MABC-2).

**C: Language function:** Vocabulary comprehension index (VCI) scores on WPPSI-IV and WISC- IV.

**D: Adaptive function:** General adaptive scores on the Adaptive Behavior Assessment System, Second Edition (ABAS-II)
